# ^40^Ar/^39^Ar dating of Glacial Termination VI: constraints on the duration of Marine Isotopic Stage 13

**DOI:** 10.1038/s41598-017-08614-6

**Published:** 2017-08-21

**Authors:** Fabrizio Marra, Fabio Florindo, Brian R. Jicha

**Affiliations:** 1 Istituto Nazionale di Geofisica e Vulcanologia, Rome, Italy; 2 Istituto di Geologia Ambientale e Geoingegneria, CNR, Rome, Italy; 3 Department of Geoscience, University of Wisconsin-Madison, Wisconsin, USA

## Abstract

We present four new ^40^Ar/^39^Ar ages of tephra layers from an aggradational succession (Valle Giulia Formation) near the mouth of the Tiber Valley in Rome that was deposited in response to sea-level rise during Marine Isotopic Stage (MIS) 13. These new ages, integrated with seven previously determined ages, provide the only extant independent, radioisotopic age constraint on glacial termination VI and on the duration of MIS 13 sea-level rise. The new geochronologic constraints suggest a long duration for the period of sea-level rise (533 ± 2 through 498 ± 2 ka) encompassing two consecutive positive peaks of the δ^18^O curve (substages 13.3 and 13.1). Consistently, the litho-stratigraphic features of the sedimentary record account for two aggradational phases separated by an intervening erosional phase. Moreover, the ages obtained for this study give us the opportunity to compare the timing of the sea-level fluctuations inferred from the stratigraphic record and that provided by the astrochronologic calibration of the Oxygen isotopic curves, and to assess the calibrations of ^40^Ar/^39^Ar standards. Results of this comparison indicate that the best match is for an age of 1.186 Ma for the Alder Creek Rhyolite sanidine and 28.201 Ma for the Fish Canyon Tuff sanidine.

## Introduction

**Figure 1:**
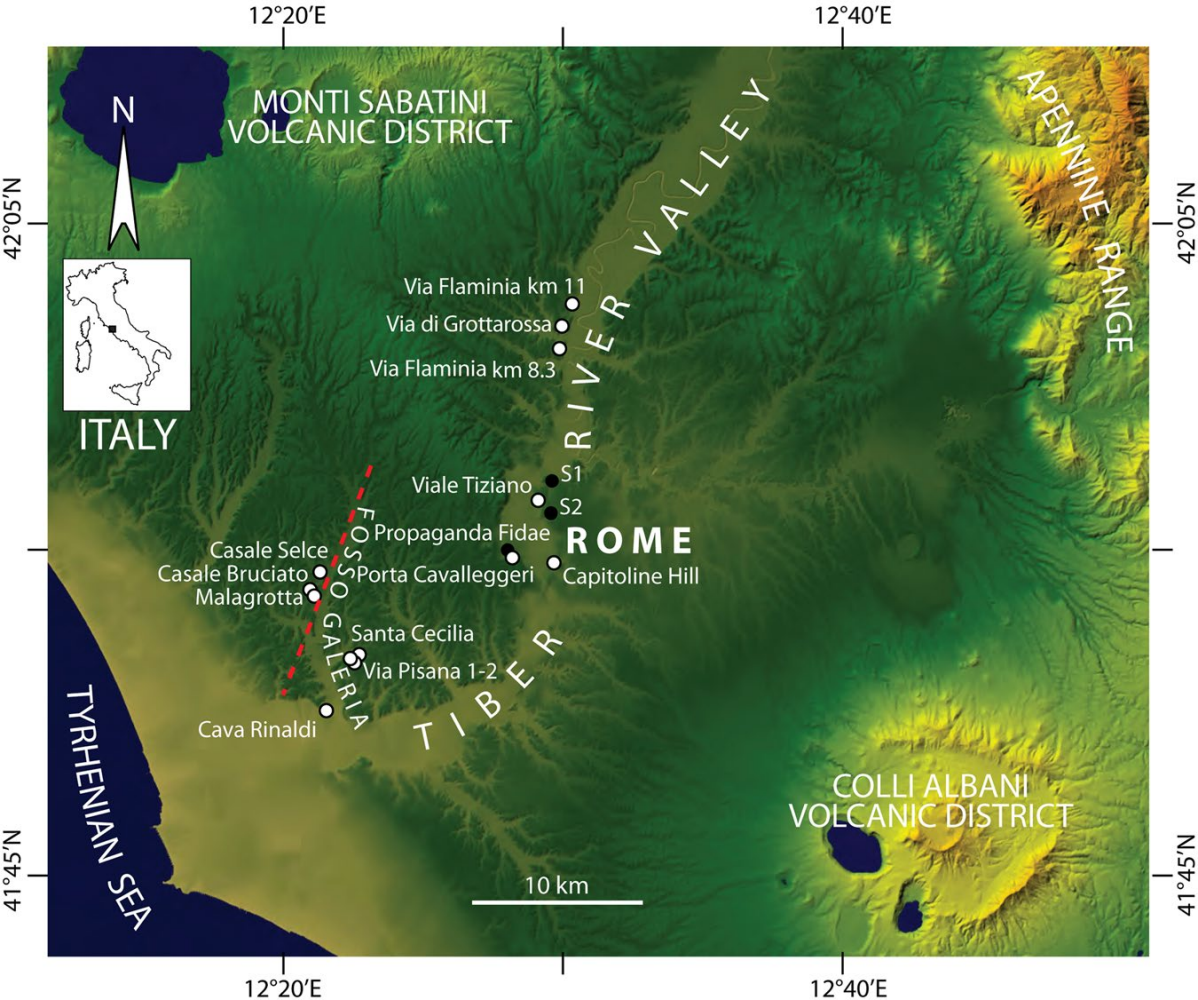
Digital Elevation Model (DEM) showing the investigated area of the Tiber River Valley and the Tyrrhenian Sea coast. Modified after TINITALY/01 square WA 6570, used with permission of the Istituto Nazionale di Geofisica e Vulcanologia, Rome. Locations of the investigated sections and interpreted boreholes are shown (open and filled circles, respectively).

Assessing ages of sedimentary successions deposited in near-coastal environments in response to sea-level fluctuations is a straightforward mean to investigate the timing of glacio-eustatic cycles and provide insights on their forcing mechanisms. In this light, the coastal plain of the Tiber River near Rome (Fig. 1) is a natural laboratory for investigating the timing of the Pleistocene glacial terminations, thanks to the presence of two active volcanic districts, yielding continuous intercalation of tephra layers within the fluvial and coastal sediments. ^40^Ar/^39^Ar dating of these tephra provides precise geochronologic constraints on the sedimentary successions and supports a strict glacio-eustatic forcing on their deposition^1–6^. An implemented conceptual model of aggradational succession has been developed^7, 8^, which accounts for the substantial synchronicity between the deposition of the coarse-grained, basal portion of the sedimentary sequence and the onset of the glacial termination. Through several ^14^C age constraints on the aggradational succession of the modern Tiber River, (refs 8 and 9) have shown that the accumulation of a several meters-thick basal gravel layer within the Tiber valley occurred since 15.1 ± 0.1 ka, and that the abrupt sedimentologic transition to a several tens of meters-thick sandy clay package of sediments occurred synchronously in a 30 km-long terminal tract of the river channel, between 13.6 ± 0.2 and 12.8 ± 0.1 ka.

Based on the correspondence of this time span with the occurrence of Meltwater-pulse 1A (14.5–13.6 ka (refs 10 and 11)), (ref. 8) used the ages of the gravel-clay transitions in the older aggradational successions of the Paleo-Tiber River, determined through ^40^Ar/^39^Ar dating of intercalated tephra layers, as a proxy of the last four glacial terminations II through V. These ages were then compared to the astrochronologically calibrated ages (e.g. ^12^) and those provided by the independently dated Relative Sea-Level (RSL) curve^13^. A very good match was found between the timing of gravel-clay transition and that of the glacial termination provided by the isotopic record of (ref. 12) for the aggradational successions of the Paleo-Tiber River deposited in response to sea-level rise during Marine Isotopic Stage (MIS) 5.5, MIS 7.5 and MIS 11 (Epi-Tyrrhenian, Vitinia, and San Paolo Formations^3, 14, 15^). In contrast, an early aggradation attributed to MIS 9 preceded the astronomical age of glacial termination IV by about 15 ka (Aurelia Formation^3, 8^). However, comparison with the RSL curve showed correspondence between this early aggradational phase as well as other minor aggradational phases within the sedimentary successions of the Paleo-Tiber River, with peaks of sea-level rise, demonstrating the high sensibility of this proxy to record even relatively small sea-level fluctuations.

Remarkably, no independent age constraint exists in the literature for glacial termination VI, apart from the ^40^Ar/^39^Ar ages by the Paleo-Tiber River aggradational successions^3, 7^, because the age of this glacio-eustatic event falls near the limit of the time interval available for U-series dating methods (ca. 500 ka). In this paper we combine four new ^40^Ar/^39^Ar ages with seven previously published ages^2, 16^, to constrain the timing of the aggradational succession (Valle Giulia Formation^3^) deposited in response to sea-level rise during MIS 13 (Table 1). In addition, we have corroborated petrographic observations with geochemical data on fourteen outcrop samples in order to provide age constraints on key volcanic deposits that are not directly dated, correlate different geologic sections, and make comparisons with published compositions for various eruptive units (Supplementary Table 1 in Supplementary information file). To accomplish this, we employed a method relying on the ratio of immobile elements (i.e. Zr/Y vs Nb/Y)^17^, that was successfully applied in several archaeological and tephrostratigraphic contexts^18–22^, demonstrating the potential to classify even deeply altered products.

**Table 1:** Summary of new and published ^40^Ar/^39^Ar ages from the Valle Giulia Formation.

Sample	Unit - Section	ACs 1.186 Ma		FCs 28.201 Ma		Reference
		Age (ka)	±2σ full	Age (ka)	±2σ full	
C5-SC	Fall C? - Santa Cecilia	461,0	±2,1			This study*
CR6-B	Fall A2 upper - Cava Rinaldi			496	±9	16*
CR5	Fall A2 - Cava Rinaldi			499,2	±5,0	16
NCR-1	Fall A1 upper - Cava Rinaldi	498,1	±1,6			This study*
C2-SC	Fall A1 upper - Santa Cecilia	495,3	±2,5			This study*
R93–22C	Fall A1 upper - Cava Rinaldi	500	±6			2, recalc. in ref. 1
CR1	GRPS** - Cava Rinaldi			510,2	±4,1	16
SAX-01	GRPS-b - Via Flaminia			511	±9	24, recalc. in ref. 16
NCR-4	TGPP - Cava Rinaldi	515,7	±1,3			This study*
LAB-01	TGPP - Via Flaminia			517	±5	24, recalc. in ref. 16
10803	TP (PDVF) - Via Flaminia			531	±2	24, recalc. in ref. 16
10803bis	TP (PDVF) - Via Flaminia			533	±2	6, recalc. in this study

^*^Data obtained at the University of Wisconsin-Madison. All other published data obtained at Berkeley Geochronology Center.

^**^Previously interpreted as TGPP^16^.

2σ full includes analytical and systematic (i.e. decay constant) uncertainties.

TP: Tufo del Palatino; PDVF: Peperino della Via Flaminia; TGPP: Tufo Giallo di Prima Porta; GRPS: Grottarossa Pyroclastic Sequence.

By using ages of four selected tephras interbedded within the aggradational succession, we provide independent time constraints with analytical uncertainties on the order of ±2 ka (2σ) on the associated sea-level fluctuations during MIS 13 and on glacial termination VI. Moreover, the newly provided ages and those of the Paleo-Tiber River dataset give us the opportunity to compare the match between the timing of the sea-level fluctuations inferred from the stratigraphic record and that provided by the astrocalibration of the isotopic curves, and to assess the calibrations of the ^40^Ar/^39^Ar standards. Note that the uncertainties on the ^40^Ar/^39^Ar ages used herein include the analytical and systematic (i.e., decay constant) uncertainties when being compared to ages of an orbitally tuned timescale.

## MIS 13 aggradational succession

The Valle Giulia Formation (hereby VGF) was introduced by (ref. 23) to designate the fluvial deposits of the Paleo-Tiber River in Rome emplaced during sea-level rise of MIS 13. (ref. 2) provided geologic constraints on the VGF through ^40^Ar/^39^Ar dating of several tephra layers, allowing (ref. 3) to define a depositional range spanning 557 ± 14 through 485 ± 2 ka for the aggradational succession associated with sea-level fluctuation of MIS 14/13.

**Figure 2:**
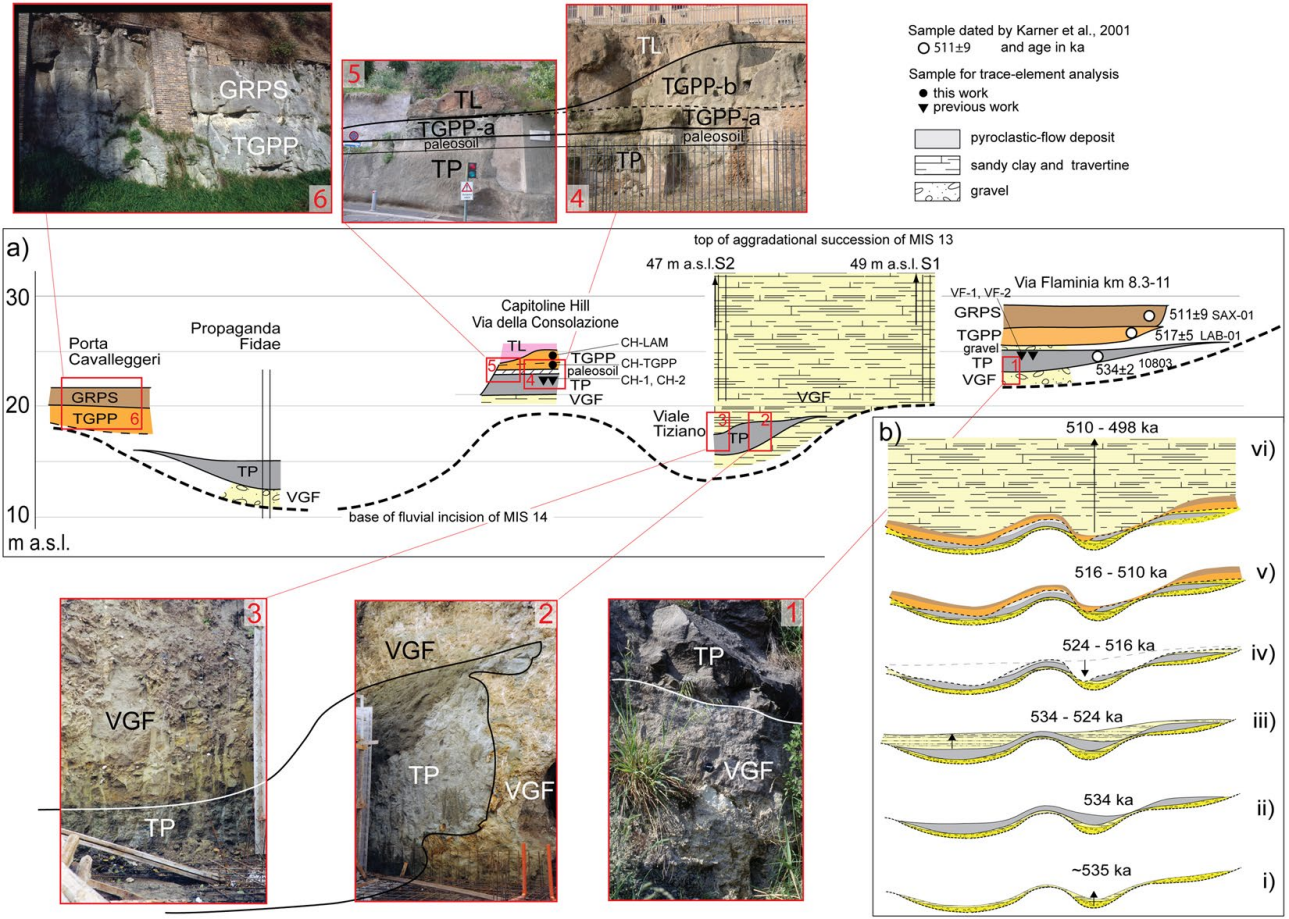
(**a**) composite N-S cross-section, correlating different outcrops and borehole logs along the Tiber valley (see Fig. 1 for location), showing the stratigraphic position of the pyroclastic-flow deposits of Tufo del Palatino (TP), Tufo Giallo di Prima Porta (TGPP) and Grottarossa Pyroclastic Sequence (GRPS)^24^, which are interbedded with the sedimentary deposits of the Valle Giulia Formation (VGF)^3^, and of Tufo Lionato (TL). (**b**) Reconstruction of the erosional/depositional phases during aggradation of the VGF, showing the occurrence of an early aggradational phase coeval with emplacement of TP (I-II-III), followed by a regression coeval with emplacement of TGPP (IV-V) and by a new aggradational phase (VI); reported ages according to this study (see text for detail).

Later on, (ref. 7) used the ^40^Ar/^39^Ar age of 528 ± 1 ka of the pyroclastic-flow deposit of Tufo del Palatino^24^ to constrain the occurrence of glacial termination VI, based on its stratigraphic position within the aggradational succession of the Valle Giulia Formation in Rome (Fig. 2). The age of this sample was recalculated to 530 ± 2 ka (2σ) in (ref. 25) (Table 1). More recently, ages of several volcanic deposits intercalated within a fluvial-lacustrine to brackish succession of the VGF cropping out at Cava Rinaldi in the Fosso Galeria stream valley (Figs 1 and 4), spanning 410 ± 4 (Grottarossa Pyroclastic Sequence - GRPS) − 496 ± 9 ka (ref. 16), provided constraints on the late aggradational phase during the high stand of MIS 13.

**Figure 3:**
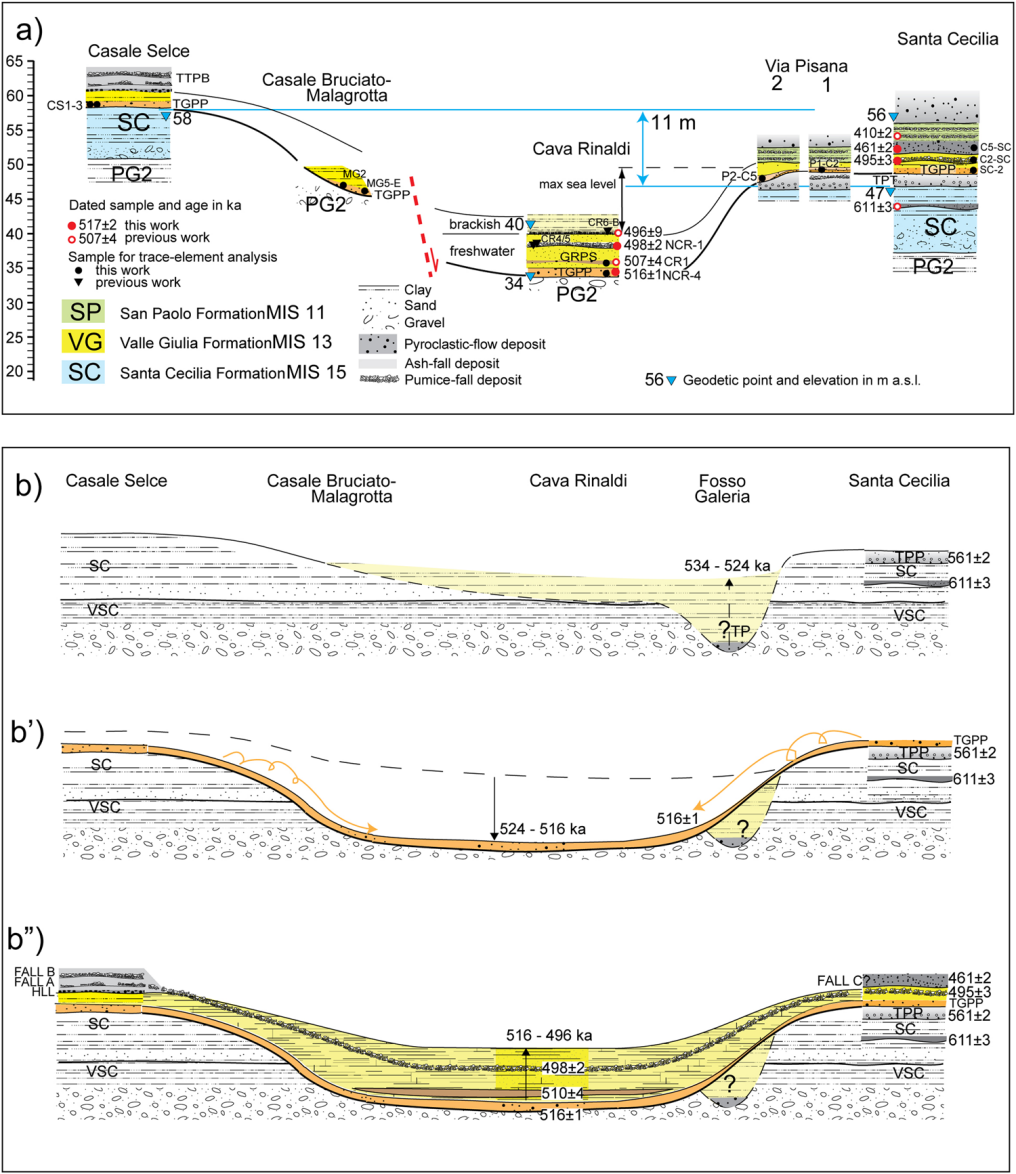
(**a**) Cross-section showing the stratigraphic relationships among the VGF and the previous (Santa Cecilia Formation -SC, MIS 15 (ref. 3)) and the following (San Paolo Formation -SP, MIS 11 (ref. 3)) aggradational succession at the investigated geologic sections in the western area of Rome (see Fig. 1 for location) b-b”) Reconstruction of the erosional/depositional phases during aggradation of the VGF showing the occurrence of an erosive phase coeval with emplacement of TGPP (b’), supposed to follow an early aggradational phase (**b**) corresponding to that evidenced in Rome to occur during emplacement of TP, and followed by deposition of the late aggradational succession of the VGF (b”) (see text for detail).

**Figure 4:**
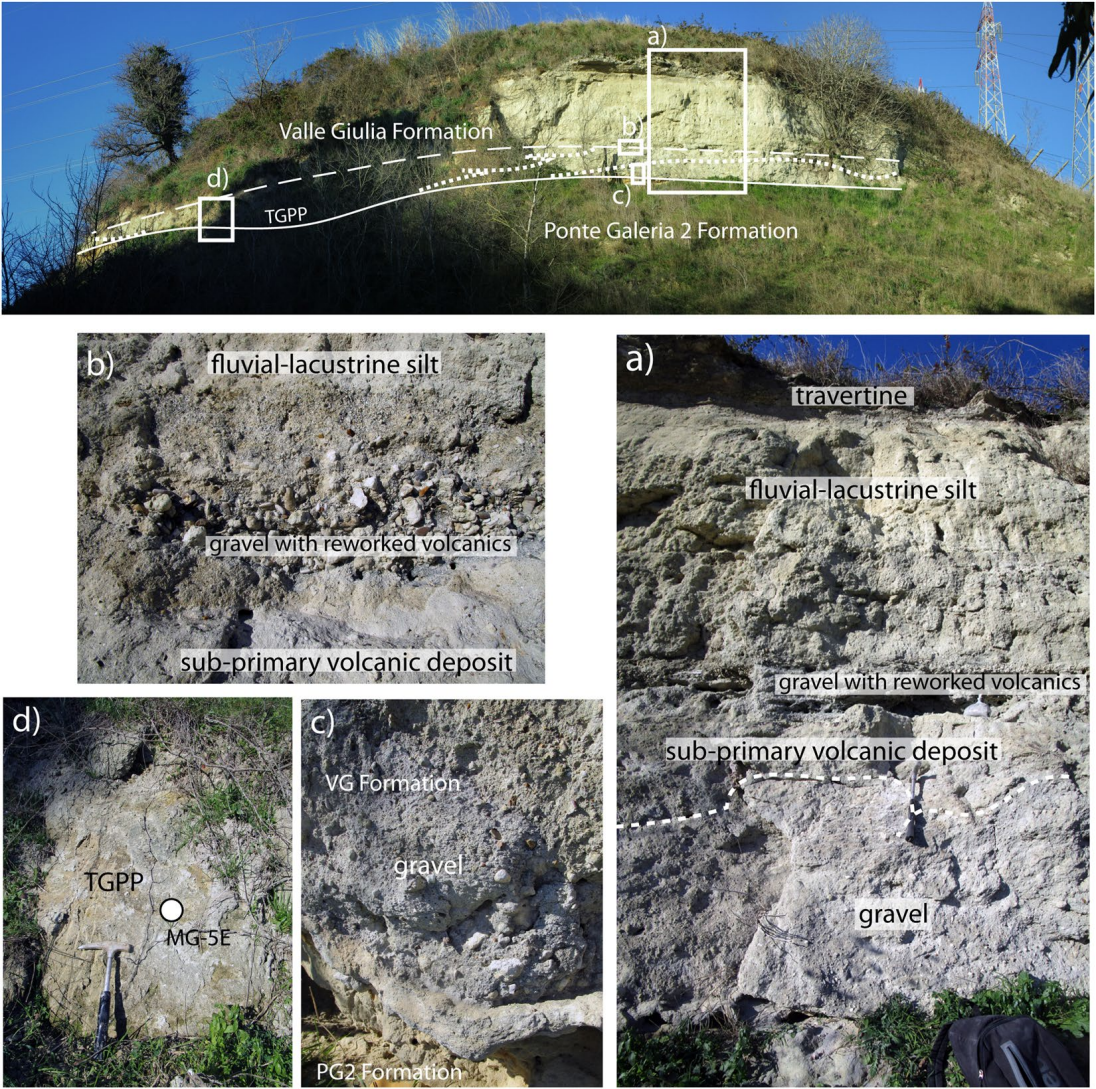
A) Malagrotta section exposing the sedimentary deposit of the Valle Giulia Formation (MIS 13), erosionally above those of the older Ponte Galeria 2 Formation (MIS 17). A massive, sand matrix supported gravel layer (**a**,**c**) is present at the base of the incision hosting the aggradational succession of the Valle Giulia Formation (MIS 13), which cut through the sand deposit of the Ponte Galeria 2 Formation (MIS 17). The gravel is laterally in contact with both the primary deposit of the TGPP (**d**), as well as with abundant, sub-primary volcanic deposit (**a**,**b**), providing further evidence that the pyroclastic-flow deposit was emplaced during an erosive phase. See text for further details.

In the present study, we have refined the geochronology at the Cava Rinaldi section through a detailed stratigraphic investigation by obtaining two new ^40^Ar/^39^Ar ages of the primary pyroclastic deposits of Tufo Giallo di Prima Porta (TGPP) and Fall A1 occurring at the base and in the middle of the fluvial-lacustrine succession (sample NCR4, NCR1, Table 1, Fig. 4). In order to provide further constraints on the deposition of the VGF, two samples of pyroclastic-fall deposits (sample C2-SC, C5-SC, Table 1) at the proximal section of Santa Cecilia (Figs 1 and 3) also have been dated and correlated with Fall A1 and Fall C, respectively. In addition, we have re-investigated and sampled for geochemical analyses all the geologic sections along the Tiber River and the Foso Galeria Stream valleys in which the VGF is exposed, in order to reconstruct sediment aggradation through time and space. By doing so, we provide quantitative constraints, both in amplitude and duration, to the sea-level fluctuations during MIS 13. ^40^Ar/^39^Ar ages performed for this study and those from literature used to constrain aggradation of the VGF are reported in Table 1. Supplementary Figure 1 shows the Zr/Y vs Nb/Y composition of the analysed samples of volcanic deposit and their correlation with known eruptive units.

## Results

### Stratigraphic evidence

The Tufo del Palatino (TP) pyroclastic-flow deposit outcrops at variable elevation throughout the area of Rome (Fig. 2). It follows channels within the paleovalley of the Tiber River, where it occurs at lower elevation with respect to the plateau-like area in the vicinity of the city center (see also Supplementary Figure 2, reconstructing the base surface of TP). These paleogeographic features suggest that the pyroclastic-flow deposit was emplaced during a marked erosional period, which caused the deep excavation of the river valleys. Moreover, at two locations within the Tiber valley incision (Via Flaminia, Fig. 2a, photo 1, and Propaganda Fidae, Fig. 2a) the TP rests directly above a thick gravel layer which constitutes the basal, coarse portion of the VGF aggradational succession in Rome. Stratigraphic evidence at Viale Tiziano and at Capitoline Hill (Fig. 2a), where TP rests on travertinaceous sand deposits of the VGF, also shows that emplacement of the pyroclastic flow occurred during the very early stages of the sea-level rise when the fine portion of the aggradational succession had just begun to accumulate laterally with respect to the fluvial channel (Fig. 2b-i-ii). When interpreted in the light of the sedimentary model by (ref. 8), these stratigraphic features substantiate the use of the TP age to date glacial termination VI at the onset of MIS 13.3 (ref. 7).

The TP pyroclastic-flow deposit appears to be partially eroded at Viale Tiziano and Via Flaminia, where the clastic deposits of the VGF unconformably overly it (Fig. 2a, and photo 2–3). At Capitoline Hill, instead, the TGPP occurs above the eroded TP. A 50 cm-thick laminated layer occurs at the base of the massive TGPP pyroclastic-flow deposit (Fig. 2a, photo 4–5, Supplementary Figure 3); geochemical signature (Supplementary Figure 1) and textural features indicate that this is a low-density, surge-flow deposit emplaced in the early stages of the eruption phase. It rests above a faintly pedogenized, altered portion of the TP pyroclastic-flow deposit (Supplementary Figure 3), evidencing the occurrence of erosion affecting the volcanic deposit and prolonged subaerial exposure.

**Figure 5:**
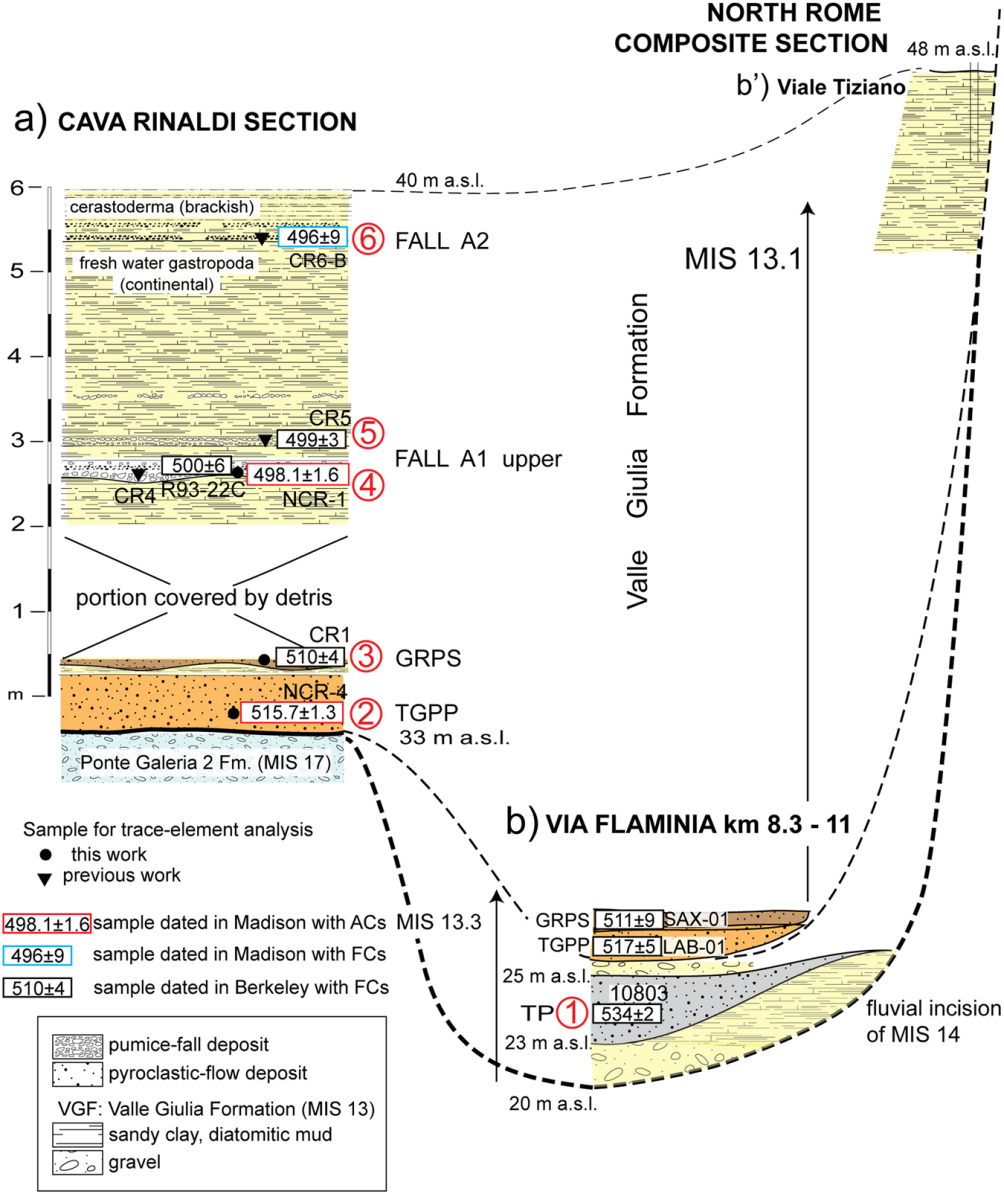
(**a**) Cava Rinaldi stratigraphy (modified and re-drawn here from previous stratigraphic scheme^16^) and correlation with a composite section merging chronostratigraphic information fron northern Rome. (**b**–b’). Positions of the six ^40^Ar/^39^Ar dated samples used in this work to constrain aggradation of the VGF (red circles, 1–6), and of other dated samples (Table 1) are shown.

These stratigraphic features favor a continued aggradational phase occurring since TP emplacement, causing incomplete filling of the paleovalleys (Fig. 2b-ii-iii). This early aggradational succession is successively truncated by a second erosive phase (Fig. 2b-iv). Evidence for this includes partial erosion affecting the TP within the Tiber Valley (i.e. Viale Tiziano), formation of a paleosoil on top of its eroded surface (i.e, Capitoline Hill), as well as by the occurrence of a gravel layer on its top (Via Flaminia km 11). Emplacement of TGPP during this renewed erosive phase (Fig. 2b–v) is suggested by its sharp contact above the eroded TP at Capitoline Hill (Fig. 2a, photo 4–5, and Supplementary Figure 3) and more clearly by stratigraphy at the Casale Selce, Via Pisana 1–2, Malagrotta, Casale Bruciato and Cava Rinaldi sections, which is described in the following paragraphs (Figs 3, 4 and 5, and Supplementary Figures 4 and 5 and 6).

The TGPP is emplaced above a marked erosive surface in Casale Selce, Malagrotta, Casale Bruciato and Cava Rinaldi (Fig. 3). The TP is never present below TGPP at these sections, suggesting its erosive removal. Moreover, the TGPP is overlain by variably thick sedimentary deposits of the VGF at the Santa Cecilia and Via Pisana 1–2 sections (Supplementary Figure 5). In Santa Cecilia, the lagoon deposits of the SCF, representing the sea-level marker for MIS 15, occur 47 m above sea level (a.s.l.), as opposed to the equivalent deposit cropping out 58 m a.s.l. in Casale Selce (Supplementary Figure 4), suggesting a tectonic dislocation of ca. 11 m between these localities^26^. In Fig. 1 we have reported the inferred trace of the fault responsible for the dislocation, based on geomorphologic evidence and on the occurrence of an equivalent offset in the borehole data from the Monte Ciocci Formation (MIS 21) in the Fosso Galeria Valley (see Fig. 5 in ref 27). The post sedimentary, tectonic dislocation^26^ is shown in the cross-sections of Fig. 3b-b”, which reconstruct the aggradational history during MIS 13 at the four investigated sections.

Evidence from Rome (Fig. 2a) suggests that the TP was emplaced within the paleoincisions forming the hydrographic network of the Fosso Galeria Stream at the onset of glacial termination VI at 533 ka, and that aggradation continued for a short interval thereafter (Fig. 3b). However, a second, marked erosive phase must have occurred before 516 ka, when the TGPP was emplaced directly upon an erosive surface, which lacks any volcanic or sedimentary deposit above it (Fig. 3b’). Moreover, the deposit of the TGPP in Via Pisana 1–2 and in Casale Bruciato displays evidence of partial reworking and rapid re-deposition (see Supplementary Figures 5 and 6). A markedly oxidized, orange, pyroclastic deposit has been observed above the gray, primary pyroclastic-flow deposit of TGPP in the Via Pisana 1 and 2 sections (Supplementary Figure 5). In Malagrotta, abundant reworked pyroclastic material of the TGPP occurs within a 10 cm-thick gravel layer, which is 20 cm above the primary pyroclastic-flow deposit (Fig. 4b). Here, a massive, sand matrix supported gravel layer (Fig. 4c) is present at the base of the incision hosting the VGF aggradational succession, and is laterally in contact with both the primary deposit of the TGPP (Fig. 4d), as well as with abundant, sub-primary volcanic deposit (Fig. 4a,b), providing further evidence that the pyroclastic-flow deposit was emplaced during an erosive phase. Stratigraphy of the Malagrotta outcrop supports the occurrence of a continued erosive phase and partial re-working after emplacement of TGPP, followed by a new aggradational phase, as evidenced by the second, thin gravel layer (Fig. 4b) covered by the upper, thick portion of the aggradational succession (Fig. 4a).

The final stages of aggradation of the GF are well documented and temporally constrained by ^40^Ar/^39^Ar ages on five tephra layers interbedded with the sedimentary deposits in Cava Rinaldi (Figs 3 and 5), four of which were dated previously^16^. The basal pyroclastic-flow deposit has been ^40^Ar/^39^Ar dated at 516 ± 1 ka in the present study (Table 1), providing an indistinguishable, but more precise age constraint for the TGPP than the 514 ± 6 ka date for the deposit cropping out in Grottarossa^24^. The very well-constrained age of 498 ± 2 for upper Fall A1 (sample NCR-1), is in good agreement with the previous age of 500 ± 6 ka, and consistent with that of 499 ± 3 ka for the immediately overlying pumice fall, provided in (ref. 16). All these ages, along with the less precise one of 496 ± 9 ka on sample CR6-B^16^, constrain the final stages of aggradation for the VGF, suggesting an age around 496 ka for the highstand of MIS 13. Additional evidence of the timing of the MIS13 highstand is provided by the occurrence of near-coast deposit (*Cerastoderma edule* bearing clay, Fig. 5a) immediately above sample CR6-B.

## Discussion

### Constraints on sea-level fluctuations

**Figure 6:**
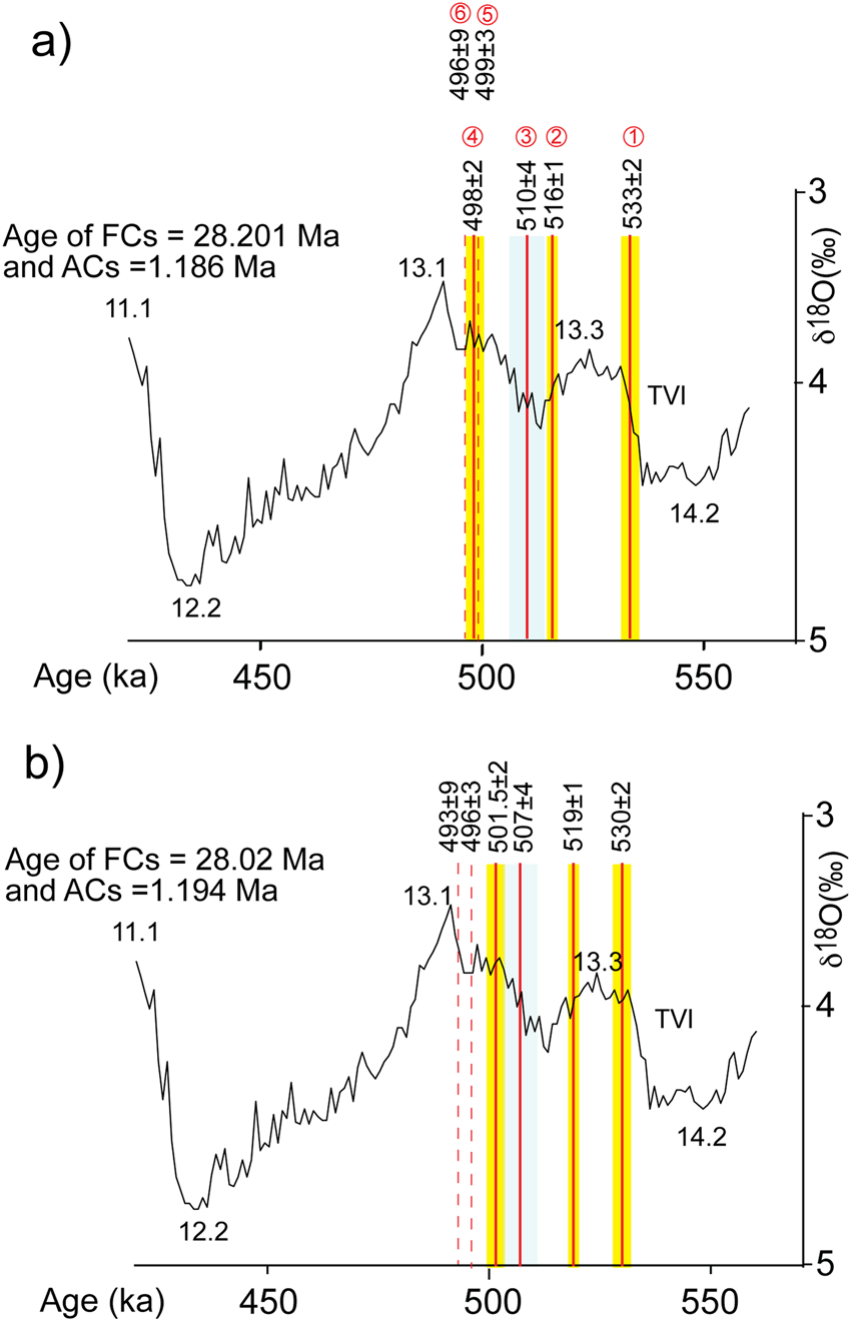
Position of the sample ages (1–6 in Fig. 5) on the astrocalibrated δ^18^O curve^12^, according to different calibrations of the standards (see text for comments). Red lines are the weighted mean ages of the dated samples (dashed lines represent ages providing no significant constraint). Yellow and grey boxes are the 2σfull uncertainties.

The base-level of the VGF exposed in northern Rome (Fig. 5b) provides evidence of the occurrence of glacial termination VI, and the basal gravel layer and the overlying fine-grained sediments were likely emplaced at the onset of MIS 13.3. The Tufo del Palatino is deposited within a fluvial channel incised in this early aggradational succession, constraining the beginning of sea-level rise to 533 ± 2 ka (see also Fig. 6).

The base-level of the VGF exposed in Cava Rinaldi (and at the other sections in western Rome shown in Fig. 3), combined with the occurrence of a gravel layer between TGPP and TP at Via Flaminia km 11, represents an intervening, new erosional phase, corresponding to sea-level fall of MIS 13.2 (Fig. 5b). The timing of maximum sea-level fall is constrained by the emplacement of TGPP at 516 ± 2 ka. An almost immediate sea-level rise since this time, at the onset MIS 13.1, is supported by the ubiquitous preservation of the TGPP, the fast accumulation of sediment above it, and the ^40^Ar/^39^Ar date of 510 ± 4 ka for the GRPS pyroclastic-flow deposit which caps the sediment accumulation within the aggradational succession in Cava Rinaldi. Finally, continued aggradation during MIS 13.1 is constrained between 498.1 ± 1.6 ka and 496 ± 9 ka, whereas the maximum sea-level rise is reached shortly after 496 ± 9 ka (Fig. 5a).

### Geochronologic constraints to the δ^18^O record and ^40^Ar/^39^Ar standard age calibrations

Based on these stratigraphic observations, the emplacement of Tufo del Palatino and of Tufo Giallo di Prima Porta pyroclastic-flow deposits occurred shortly after and shortly before, respectively, the climax of two successive erosional phases. This datum, combined with the ^40^Ar/^39^Ar ages of these volcanic deposits, is consistent with the occurrence of two consecutive sea-level oscillations, evidenced by the two peaks of the δ^18^O curve corresponding to sub-stages 13.3 and 13.1 (Fig. 6).

However, when the ^40^Ar/^39^Ar ages of TP and TGPP are assessed for the different age calibrations proposed for the ACs and FCs standards^28–32^, they assume a different position in the δ^18^O curve, which may be more or less consistent with the climatic implication of the stratigraphic record. In particular, assuming a negligible phase lag between the timing of the sea-level rise and the subsequent increase in the δ^18^O, a fact that is clearly supported by comparison among the relative sea-level curve from the Red Sea indicators^13^, the benthic δ^18^O record^12^, and the age constraints on glacial terminations provided by the Paleo-Tiber River aggradational successions^8^, the age of TP and TGPP must fall immediately before and after, respectively, the negative peaks of MIS 14.2 and 13.2 (Fig. 6a).

Notably, ages provided by calibration assumed in this paper for FCs (28.201 Ma)^28^ and ACs (1.186 Ma)^31^ match well with astrocalibration of the δ^18^O curve by (ref. 12). A recalculated age of 533 ± 2 ka for TP falls exactly on the rising portion of the curve corresponding to glacial termination VI, which in (ref. 12) is dated also at 533 ka. Similarly, an age of 516 ± 2 ka for TGPP falls at the end of the decreasing portion of the curve, shortly before MIS 13.2, while that of 510 ± 4 for GRPS matches the subsequent rising tract, leading to peak of MIS 13.1 (Fig. 6a).

In contrast, when the age of 1.194 Ma for ACs^28, 29^ is assumed, an evident misfit between the climatic implication of TGPP and its position in the δO^18^ curve is observed (Fig. 6b). Indeed, an age of 519 ± 2 ka predates the sea-level fall of MIS 13.2, conflicting with the evidence of the occurrence of a marked erosive phase preceding emplacement of TGPP, which matches the late highstand of MIS 13.3. We also remark that using the calibration at 28.02 Ma for FCs would cause the TP age to correspond with the highstand of MIS 13.3, in evident conflict with the emplacement of this pyroclastic-flow deposit within a deeply incised fluvial valley.

## Conclusions

New geochronologic and sedimentary data presented here show that the stratigraphic record of the VGF represents among the best records of glacial termination VI and the associated sea-level fluctuations during MIS 13. In particular, the age of 533 ± 2 ka for the Tufo del Palatino, combined with its stratigraphic position immediately above the basal coarse gravel layer of the VGF, confirms the sedimentologic conceptual model proposed in (ref. 8), after which the gravel-clay transitions in the aggradational successions of the Paleo-Tiber River is coeval with the glacial terminations. Consistent with the VGF record, the astrochronologic age of glacial termination VI is 533 ka (ref. 12).

Moreover, the 516 ± 2 ka TGPP has a stratigraphic position above a marked erosional surface and is partially re-worked and re-deposited, all of which correspond well to its emplacement during an interstadial sea-level fall separating sub-stages 13.3 and 13.1, whose negative peak on the δ^18^O curve is dated at 513 ka (ref. 12).

Finally, the age of 510 ± 4 ka for the GRPS, combined with its stratigraphic position above ca. 1 m of fine sediment overlying the TGPP, and the age of 498 ± 2 ka for the pumice fallout intercalated in the upper portion of the fluvial-lacustrine deposits immediately below the transition to lagoonal sediments, are consistent with renewed sea-level rise by 512 ka at the onset of MIS 13.1 with a maximum sea-level reached by 495 ka on the δ^18^O curve (ref. 12).

The sedimentary conceptual model for the VGF presented in this paper can be applied when investigating the timing of past glacial terminations and glacio-eustatic sea-level fluctuations of any amplitude globally, especially in regions in which the combined geographic (a fluvial network close to the coast, providing a sedimentary record of aggradational successions) and geologic (a volcanic area active in the past 1 Ma, providing datable material) predisposing factors are present, like in most continental plate margins.

## Methods

### ^40^Ar/^39^Ar data

In order to constrain aggradation of the sedimentary deposits of the Valle Giulia Formation, we combine four new and one published ^40^Ar/^39^Ar ages performed at the WiscAr Laboratory at the University of Wisconsin-Madison with six previously published^2, 16, 24^ ages obtained at the Berkeley Geochronology Center (Table 1). These latter eight age determinations were measured using older calibrations for the Fish Canyon Tuff sanidine (FCs) and Alder Creek Rhyolite sanidine (ACs)^2, 16, 24^. Several recent studies have indicated that the age of the Alder Creek Rhyolite sanidine is ~1.185–1.186 Ma (refs 30–32). Thus, we have recalculated the eight published ages using recent standard age calibrations (i.e. ACs = 1.186 Ma; FCs = 28.201 Ma; refs 31 and 33 and the decay constant of ref. 34) for comparative purposes (Table 1). For all samples, the youngest population of single crystal fusion dates is interpreted to reflect the eruption age of the sample. Full analytical data are provided in Supplementary Dataset #1.

### Geochemical data

The most common geochemical methods of classification for volcanic rocks (e.g., electron microprobe glass geochemistry) can be applied only to unaltered deposits. In order to classify the strongly weathered pyroclastic flow deposits cropping out at Casale Selce, Casale Bruciato, Malagrotta, Santa Cecilia and Via Pisana 1–2 localities, and to provide a comparative dataset with those cropping out and dated at Cava Rinaldi, we have measured and used selected immobile trace-element compositions.

Thirteen bulk samples of volcanic deposits collected at the geologic sections mentioned above (Supplementary Table 1) were analyzed for major and trace element composition during four different laboratory runs at Activation Laboratories, Canada by Lithium Metaborate/Tetraborate Fusion ICP-MS. The fused samples were diluted and analyzed by Perkin Elmer Sciex ELAN 6000, 6100 or 9000 ICP/MS. Three blanks and five controls (three before the sample group and two after) were analyzed for each group of samples. Wet chemical techniques were used to measure the loss on ignition (LOI) at 900 °C. International rock standards have been used for calibration and the precision is better than 5% for Rb and Sr, 10% for Ni, Zr, Nb, Ba, Ce, and La, and 15% for the other elements.

Full data are reported in Supplementary Dataset #2, results are summarized in the discrimination diagram of Supplementary Figure 1, where Zr/Y vs Nb/Y compositions of the analyzed samples are compared to those from literature pertaining to the volcanic rocks of Monti Sabatini and Colli Albani districts.

## Electronic supplementary material

Supplementary File

Dataset 1

Dataset 2
