# Supplementary material for: 40Ar/39Ar dating of Glacial Termination VI: constraints on the duration of Marine Isotopic Stage 13
Source: Sci Rep. 2017 Aug 21;7:8908. doi: 10.1038/s41598-017-08614-6 (PMC5566960; doi:10.1038/s41598-017-08614-6)
Supplement: Supplementary file 1 — Supplementary File [file 41598_2017_8614_MOESM1_ESM.pdf]

$^{40}Ar/^{39}Ar$ dating of Glacial Termination VI: constraints on the duration of Marine Isotopic Stage 13

Fabrizio Marra[1], Fabio Florindo[1], Brian R. Jicha[2]

[1] Istituto Nazionale di Geofisica e Vulcanologia, Rome, Italy
[2] Department of Geoscience, University of Wisconsin-Madison, USA

Corresponding Author: Fabrizio Marra
Istituto Nazionale di Geofisica e Vulcanologia, Via di Vigna Murata 605, 00143 Rome, Italy
e-mail: fabrizio.marra@ingv.it

Supplement Data File #1

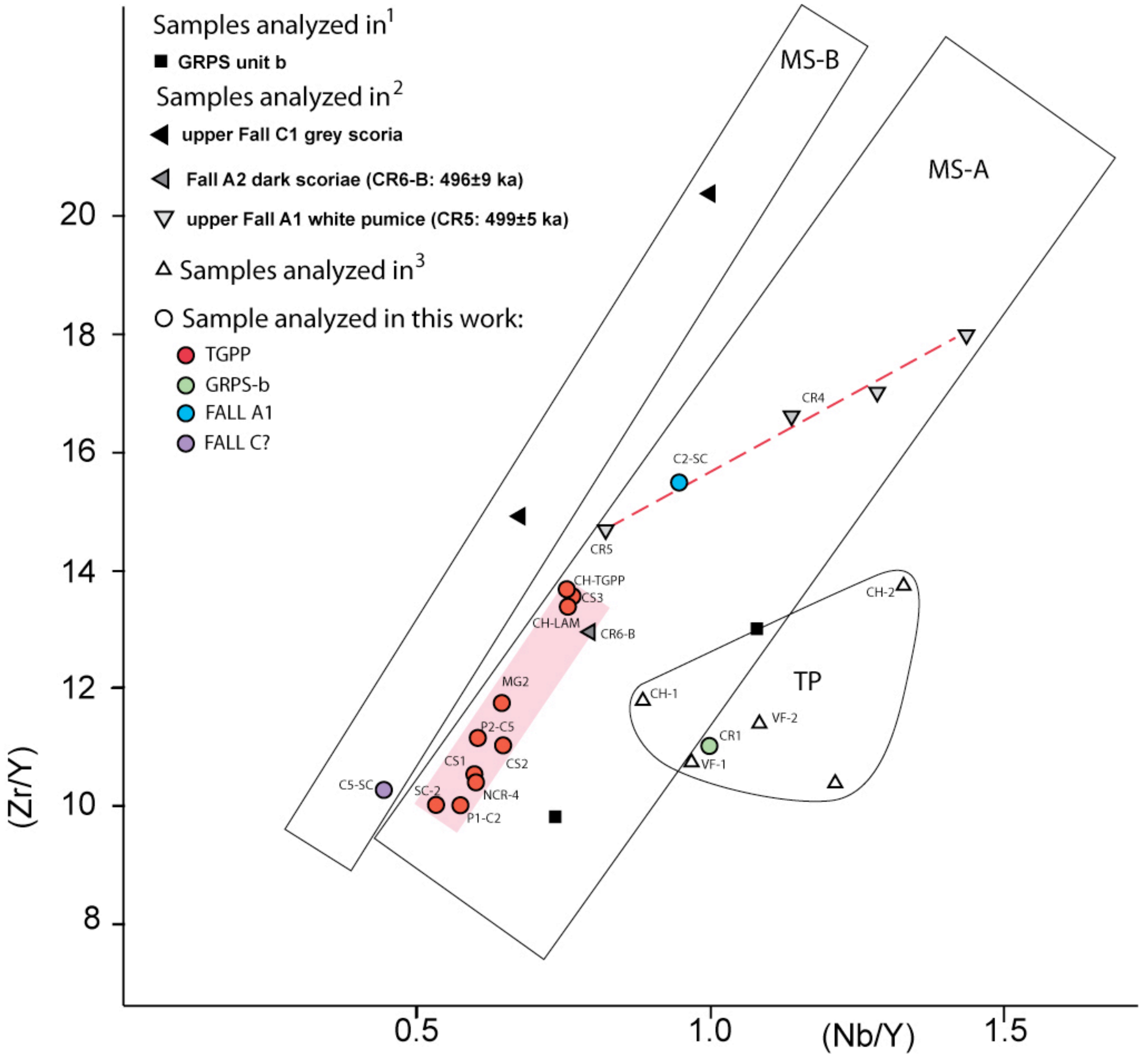

Supplementary Figure 1 - Zr/Y vs Nb/Y discrimination diagram

Samples attributed to Tufo Giallo di Prima Porta (TGPP) (red circles) cluster within a narrow compositional field (red shaded area) around the TGPP sample dated in the present study at 515.7±1.3 ka (NCR-4), and are clearly distinguished with respect to other volcanic products analyzed in recent literature. MS-A and MS-B: main compositional fields for the products of the Monti Sabatini discrict defined in[1]. TP: Tufo del Palatino; GRPS: Grottarossa Pyroclastic Sequence. Stratigraphic position of the samples is shown in the cross-sections and in the pictures of Figure 2, 3, and 4, and in the Supplementary Figures 2, 3, 5, 6.

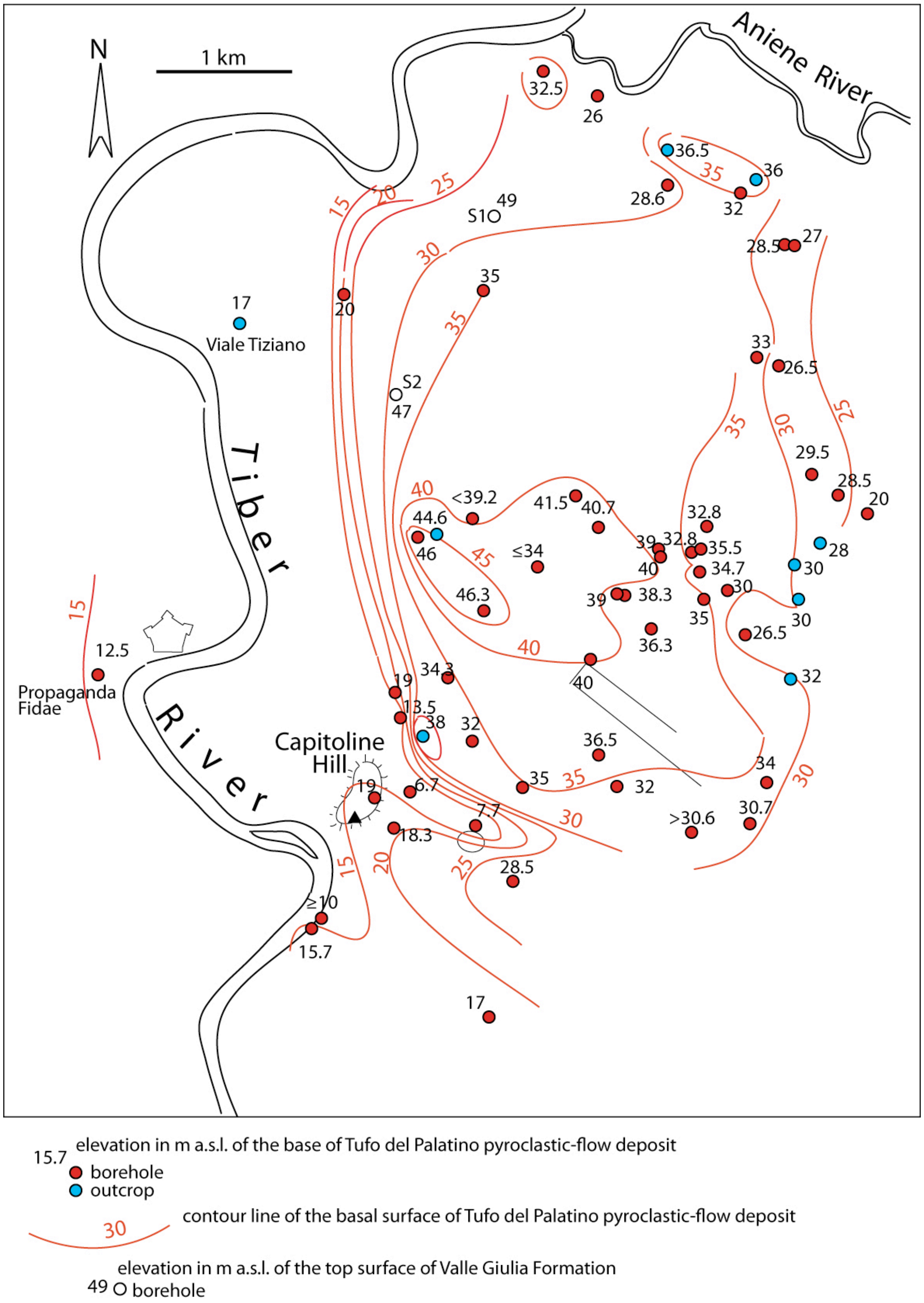

Supplementary Figure 2 - Base of Tufo del Palatino pyroclastic-flow deposit

Reconstruction of the basal surface of the Tufo del Palatino pyroclastic-flow deposit in central Rome, from borehole and outcrop data.
Map hand drawn with Adobe© Illustrator CS3 13.0.0 graphic program.

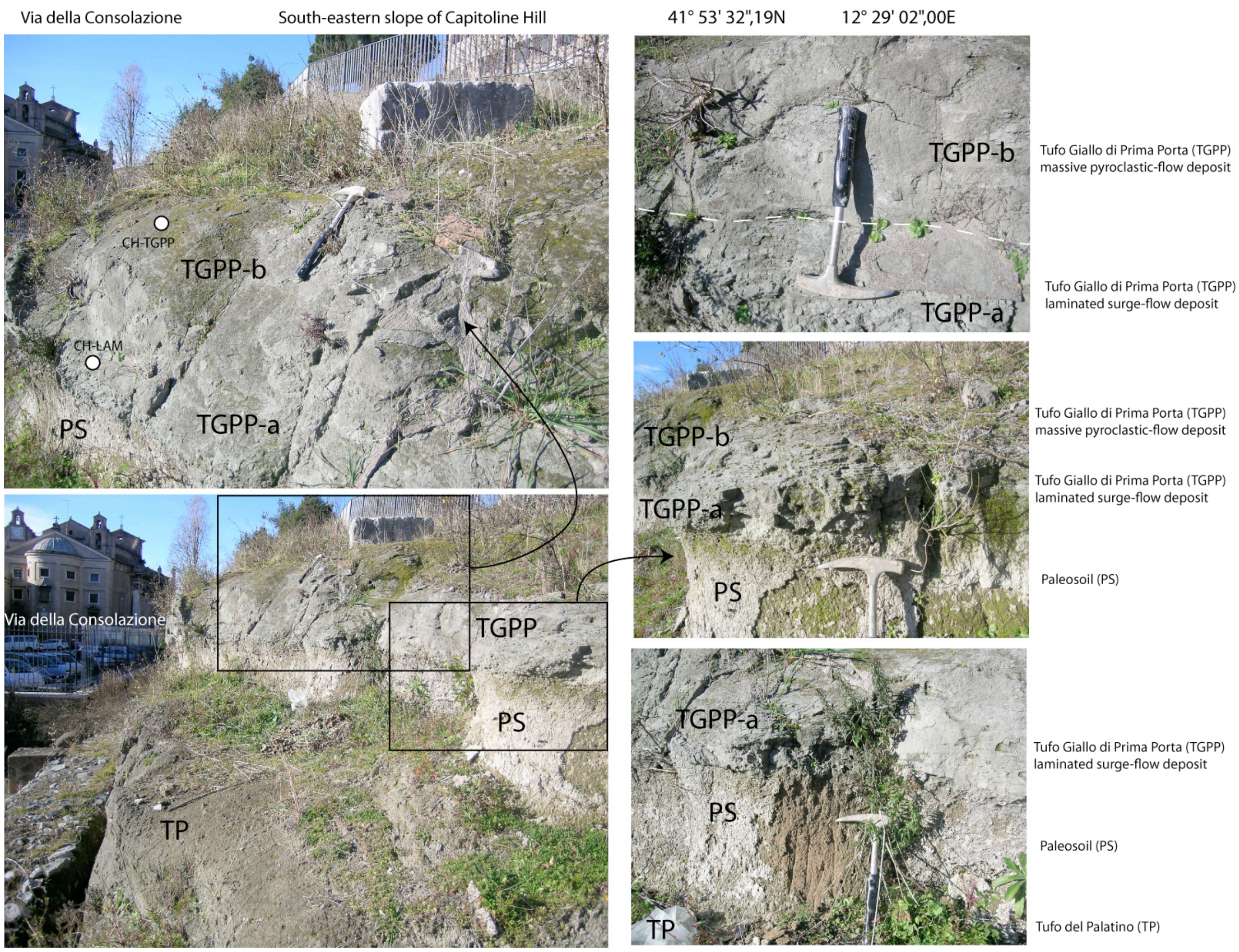

Supplementary Figure 3 - Capitoline Hill

Detail of the stratigraphy at Capitoline Hill (see Supplementary Figure 2 for location). The Tufo Giallo di Prima Porta (TGPP) occurs above the eroded Tufo del Palatino (TP). A 50 cm-thick laminated layer occurs at the base of the massive TGPP pyroclastic-flow deposit; geochemical signature (Supplementary Figure 1) and textural features indicate that this is a low-density, surge-flow deposit emplaced in the early stages of the eruption phase. It rests above a faintly pedogenized, altered portion of the TP pyroclastic-flow deposit (paleosoil), evidencing the occurrence of erosion affecting the volcanic deposit and prolonged subaerial exposure. Position of two samples of TGPP analyzed for trace-element geochemistry in the present work (CH-TGPP, CH-LAM; Supplementary Figure 1, Supplementary Table 1) is shown.

This photograph and all the other in this file are taken in public places and do not require permision to publication.

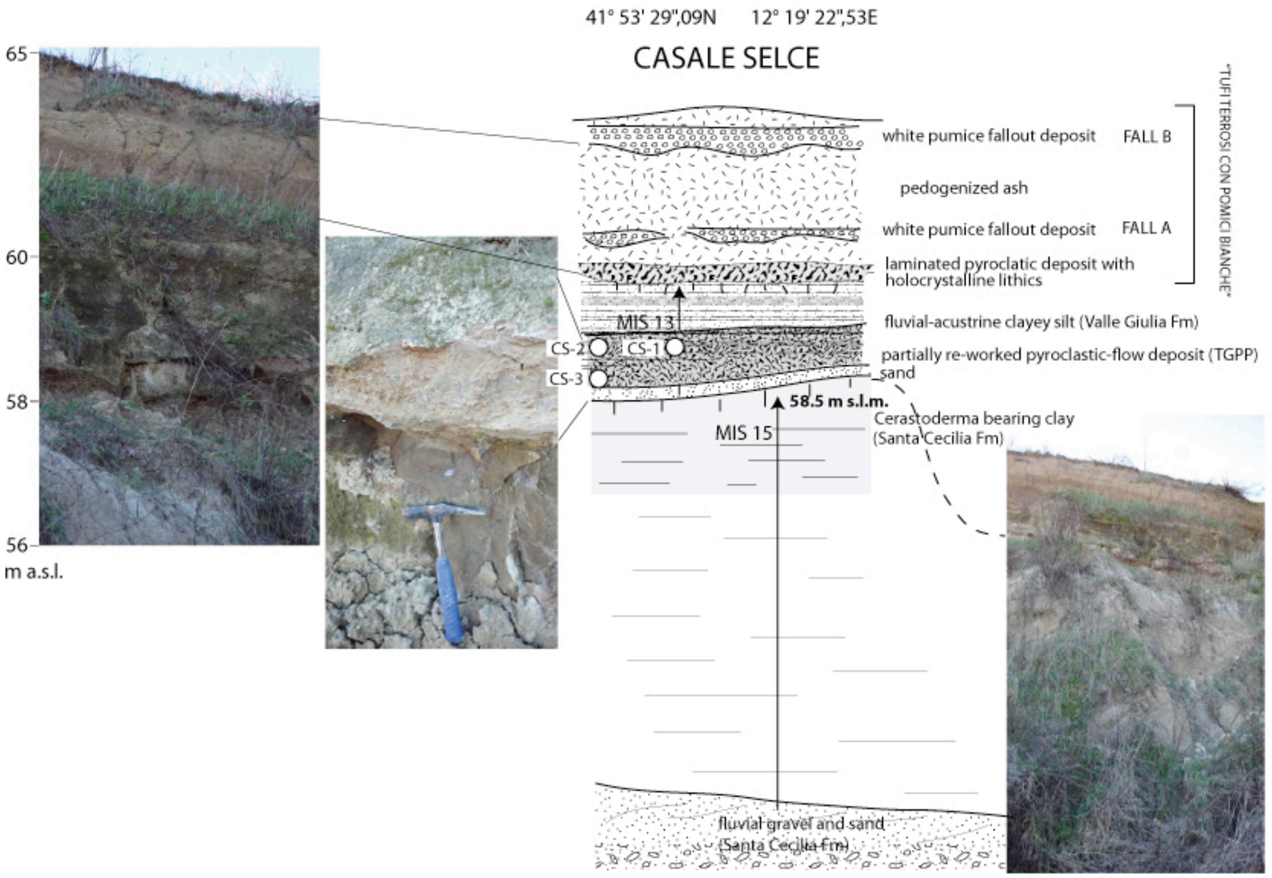

Supplementary Figure 4 - Casale Selce

Detailed stratigraphy at Casale Selce (see Figure 1 in the main text for location) exposing the pyroclastic-flow deposit of Tufo Giallo di Prima Porta (TGPP) and the overlying air-fall succession. The occurrence of the idiosynchratic "Olocrystalline lithic layer" at the base of this succession provides correlation with the Tufi Terrosi con Pomici Bianche[4], dated here at 498±2 ka (Fall A1; Supplementary Table 1) . Position of three samples of TGPP analyzed for trace-element geochemistry in the present wotk (CS-1, CS-2, CS-3; Supplementary Figure 1; Supplementary Table 1) is shown.

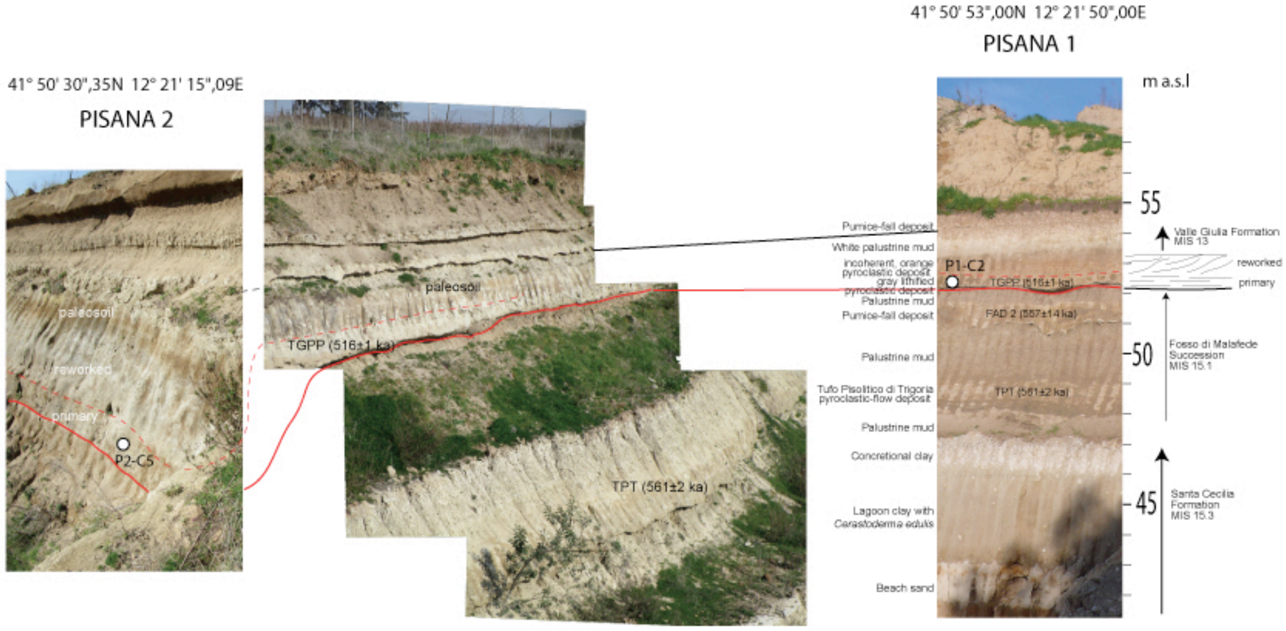

Supplementary Figure 5 - Via Pisana 1-2

Detailed stratigraphy at Via Pisana 1 and Via Pisana 2 sections (see Figure 1 in the main text for location). The deposit of the Tufo Giallo di Prima Porta (TGPP) displays evidence of partial reworking and rapid re-deposition, as evidenced by the occurrence of a markedly oxydated, orange in color, loose pyroclastic deposit above the primary pyroclastic-flow deposit . Position of two samples of TGPP analyzed for trace-element geochemistry in the present wotk (P2-C5, P1-C2; Supplementary Figure 1; Supplementary Table 1) is shown.

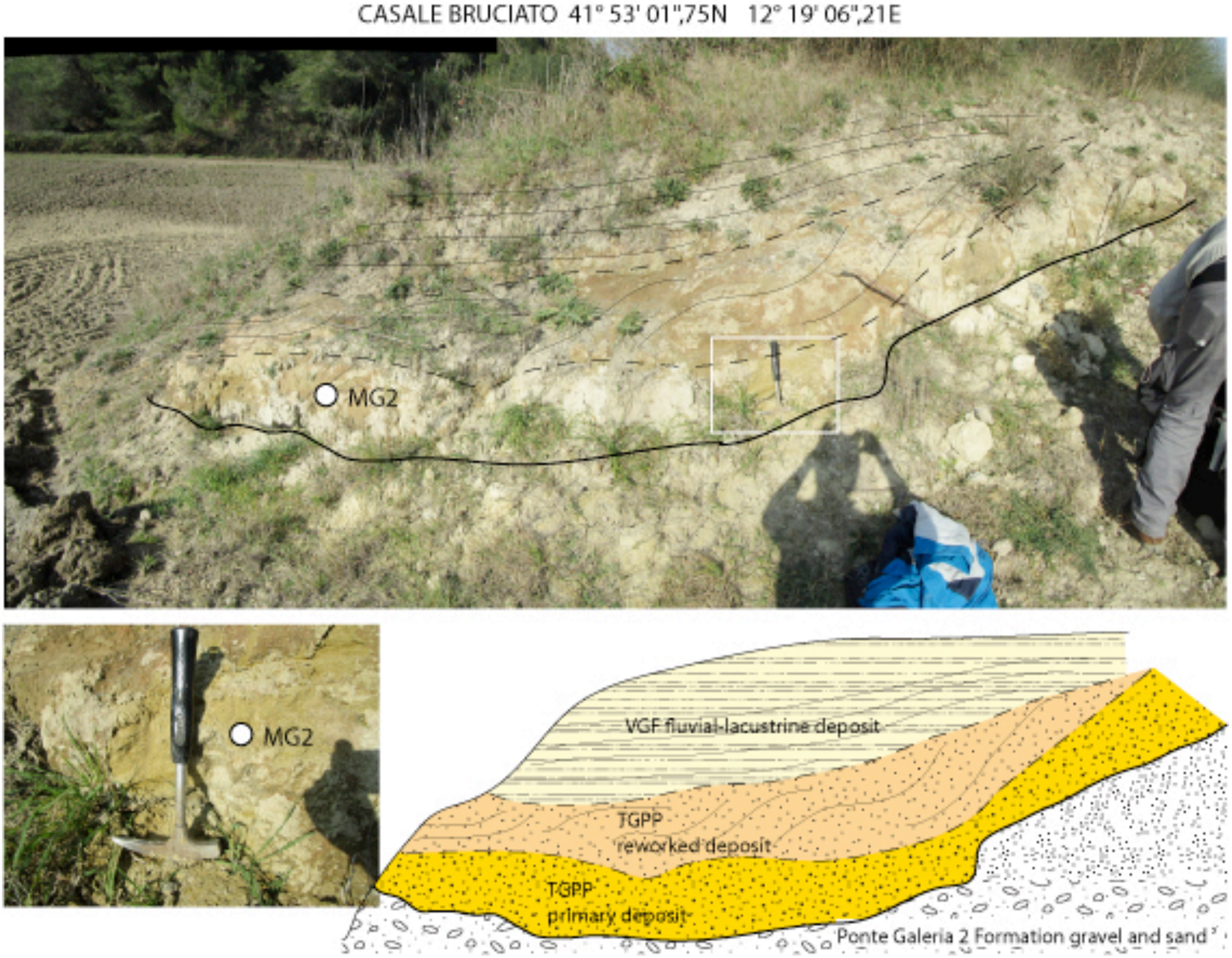

Supplementary Figure 6 - Casale Bruciato

Detailed stratigraphy at Casale Bruciato (see Figure 1 in the main text for location). The deposit of the Tufo Giallo di Prima Porta (TGPP) overlies an erosive surface cutting through the deposits of the Ponte Galeria 2 Formation (MIS 17) and displays evidence of partial reworking and re-deposition, as evidenced by the occurrence of a markedly oxydated, loose pyroclastic deposit above the primary pyroclastic-flow deposit. Position of one sample of TGPP analyzed for trace-element geochemistry in the present wotk (MG2; Supplementary Figure 1; Supplementary Table 1) is shown.

SAMPLE	UNIT	$^{40}Ar/^{39}Ar$ AGE	LOCALITY	LATITUDE	LONGITUDE
VF-1[3]	TP	533±2[4]	Via Flaminia Km 11	41° 58' 22",38N	12° 29' 31,"91E
VF-2[3]	TP		Via Flaminia Km 8	41° 57' 26",42N	12° 29' 05",31E
CH-1[3]	TP		Via d. Consolazione	41° 53' 29",81N	12° 28' 58",77E
CH-2[3]	TP		Via d. Consolazione	41° 53' 29",81N	12° 28' 58",77E
CH-LAM	TGPP		Capitoline Hill	41° 53' 32",19N	12° 29' 02",00E
CH-TGPP	TGPP		Capitoline Hill	41° 53' 32",19N	12° 29' 02",00E
CS1	TGPP		Casale Selce	41° 53' 29",09N	12° 19' 22",53E
CS2	TGPP		Casale Selce	41° 53' 29",09N	12° 19' 22",53E
CS3	TGPP		Casale Selce	41° 53' 29",09N	12° 19' 22",53E
MG2	TGPP		Casale Bruciato	41° 53' 01",75N	12° 19' 06",21E
SC-2	TGPP		Santa Cecilia	41° 50' 45",30N	12° 21' 09",00E
C2-SC	FALL A1	495±3	Santa Cecilia	41° 50' 45",30N	12° 21' 09",00E
C5-SC	FALL C?	461±2	Santa Cecilia	41° 50' 45",30N	12° 21' 09",00E
P1-C2	TGPP		Via Pisana 1	41° 50' 53",00N	12° 21' 50",00E
P2-C5	TGPP		Via Pisana 2	41° 50' 30",35N	12° 21' 15",09E
NCR-4	TGPP	516±1	Cava Rinaldi	41° 49' 30",45N	12° 19' 55",30E
CR1	GRPS	510±4[1]	Cava Rinaldi	41° 49' 30",45N	12° 19' 55",30E
CR4[1]	FALL A1	500±6[5]	Cava Rinaldi	41° 49' 30",45N	12° 19' 55",30E
CR5[1]	FALL A1	499±6 [1]	Cava Rinaldi	41° 49' 30",45N	12° 19' 55",30E
CR6-B [1]	FALL A2	496±9 [1]	Cava Rinaldi	41° 49' 30",45N	12° 19' 55",30E

Supplementary Table 1 - Samples for geochemical analyses

Samples analyzed for trace-element composition in this work and from literature, plotted in the Zr/Y vs Nb/Y discrimination diagram of Supplementary Figure 1. Ages of the samples are reported according to the standard calibrations adopted in this paper. Full geochemical analyses are provided in Supplementary Dataset #2.

Supplementary References

1. Marra, F. *et al*. Major explosive activity in the Sabatini Volcanic District (central Italy) over the 800-390 ka interval: geochronological - geochemical overview and tephrostratigraphic implications. *Quat. Sci. Rev.* **94**, 74–101 (2014).

2. Marra, F., Deocampo, D., Jasckson, M. D. & Ventura, G. The Alban Hills and Monti Sabatini volcanic products used in ancient Roman masonry (Italy): an integrated stratigraphic, archaeological, environmental and geochemical approach. *Earth-Science Reviews* **108**, 115–136 (2011).

3. Farr, J., Marra, F. & Terrenato, N. Geochemical identification criteria for "peperino" stones employed in ancient Roman buildings: a Lapis Gabinus case study. *Journal of Archaeological Science: Reports* **3**, 41–51 (2015).

4. Karner, D. B., Marra, F. & Renne, P. R. The history of the Monti Sabatini and Alban Hills volcanoes: groundwork for assessing volcanic-tectonic hazards for Rome. *J. Volc. and Geoth. Res.* **107**, 185-215 (2001).

5. Karner, D. B. & Renne, P.R. $^{40}Ar/^{39}Ar$ geochronology of Roman province tephra in the Tiber River Valley: age calibration of Middle Pleistocene sea-level changes. *Geol. Soc. Am. Bull.* **110**, 740-747 (1998).

6. Marra F., Karner, D. B., Freda, C., Gaeta, M. & Renne, P. R. Large mafic eruptions at the Alban Hills Volcanic District (Central Italy): chronostratigraphy, petrography and eruptive behavior. *Journ. of Volc. and Geoth. Res.* **179**, 217-232 (2009). doi:10.1016/j.jvolgeores.2008.11.009
